# Spread of carbapenemase-producing *Serratia* spp. in France from 2016 to 2024: a comparative genomic study

**DOI:** 10.1080/22221751.2026.2671515

**Published:** 2026-05-07

**Authors:** Inès Rezzoug, Sandrine Bernabeu, Kenza Ouacel, Aurelien Birer, Agnès B. Jousset, Gerald Larouy-Maumus, Rémy A. Bonnin, Cécile Emeraud, Laurent Dortet

**Affiliations:** a Bacteriology-Hygiene Unit, Assistance Publique-Hôpitaux de Paris, AP-HP Paris Saclay, Bicêtre Hospital, Le Kremlin-Bicêtre, France; b Team ‘Resist’ UMR1184 Immunology of Viral, Auto-Immune, Hematological and Bacterial Diseases (IMVA-HB), Faculty of Medicine, INSERM, Paris-Saclay University, Le Kremlin-Bicêtre, France; c Associated French National Reference Center for Antibiotic Resistance: Carbapenemase-Producing Enterobacterales, Le Kremlin-Bicêtre, France; d SEPSIS Comprehensive Center – IHU SEPSIS, France; e Microbes, Intestin, Inflammation et Susceptibilité de l'Hôte (M2iSH), UMR, USC INRAE, Clermont-Ferrand, France; f Centre National de Référence de la Résistance aux antibiotiques, service de Bactériologie, CHU Gabriel-Montpied, Clermont-Ferrand, France; g Department of Life Sciences, Faculty of Natural Sciences, MRC Centre for Molecular Bacteriology and Infection, Imperial College London, London, UK

**Keywords:** Carbapenemases, epidemiology, Enterobacterales, β-lactamase, *Serratia* spp, β-lactamines/β-lactamase inhibitor

## Abstract

Carbapenemase-producing *Serratia* spp. are increasingly implicated in healthcare-associated infections. This study aimed to characterize the genetic diversity, resistance mechanisms, and susceptibility to last resort antimicrobials of carbapenem-resistant *Serratia* spp. in France. From 2016 to 2024, 193 carbapenemase-producing *Serratia* spp. from France were investigated. WGS enabled species identification, MLST typing, resistome characterization and phylogenetic analysis. Antimicrobial susceptibility was assessed by broth microdilution, focusing on last resort antimicrobials. Intrinsic resistance to polymyxins was explored by genomic analysis and lipid A mass spectrometry. *S. sarumanii* (54%) and *S. nevei* (41%) dominated the epidemiology while no *S. marcescens* was identified. The most prevalent carbapenemases were OXA-48-like (56%), NDM (25%), IMP (8%), and VIM (7%). MLST identified 67 sequence types (ST) with 42% of the isolates belonging to four high-risk clones: ST-601, ST-298, ST-477, ST-600 and ST-474. β-lactamse susceptibility was low, but aztreonam–avibactam remained highly active (98%). Intrinsic resistance to polymyxin did not result from lipid A modifications as reported for *Proteae*. In France, carbapenemase-producing *Serratia* spp. were dominated by *S. sarumanii and S. nevei*. The absence of *S. marcescens*, the genus leader, highlighted the need to update the MALDI-TOF database. This study highlights *Serratia* as an underestimated reservoir of multidrug resistance.

## GRAPHICAL ABSTRACT

**Figure:**
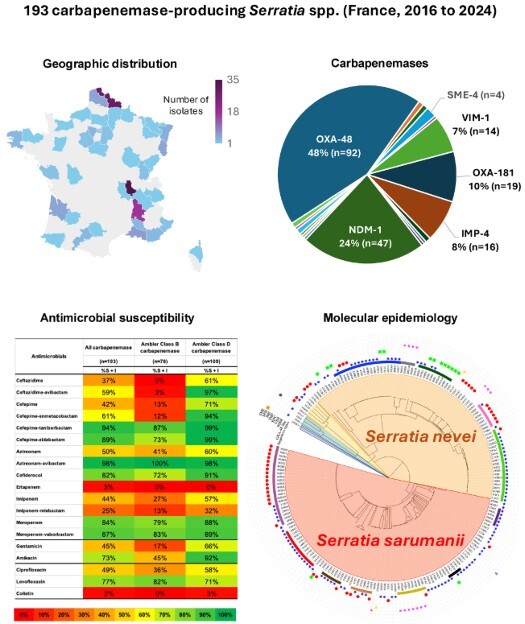

## Introduction

*Serratia* spp. were first described in 1819 and initially considered non-pathogenic organisms. For many years, they were regarded as harmless and were used as tracer organisms in medical experiments owing to their characteristic red pigment [1]. *Serratia* spp. are facultatively anaerobic, Gram-negative bacilli belonging to the Enterobacterales. In 2025, the *Serratia* genus includes 24 species according to the List of Prokaryotic names with Standing in Nomenclature (LPSN; https://lpsn.dsmz.de). These bacteria are ubiquitous in the environment and can be isolated from water, soil, and plants [1].

Their high genetic plasticity allows *Serratia* isolates to adapt to and persist in hospital environments, particularly in neonatology and intensive care units (ICUs), where they are described as a major cause of infections [2]. *Serratia* spp. have been associated with a broad spectrum of human infections, including pneumonia, bloodstream infections, meningitis, peritonitis, endocarditis, arthritis, osteomyelitis, keratitis, urinary tract infections, and skin infections [3–7].

Although more than 100 *Serratia*-related outbreaks have been reported from 1968 to 2019 [8], most of the published infections have been described as sporadic cases [9]. Moreover, a growing number of *Serratia* spp. related infections are reported worldwide, reflecting their increasing clinical significance and global spread.

*Serratia* spp. exhibit intrinsic resistance to several antimicrobials, including polymyxins (*e.g.* colistin), nitrofurantoin and macrolides. They are intrinsically resistant to most aminoglycosides, except gentamicin, due to the production of a chromosome-encoded AAC(6′) enzyme. In addition, *Serratia* is reported resistant to mecillinam because of the low affinity of their penicillin-binding protein 2 (PBP2) [10]. They also harbour a chromosome-encoded AmpC-type cephalosporinase (SRT-like enzymes), which confers resistance to amoxicillin and to first- and second-generation cephalosporins. Accordingly, the treatment of *Serratia* infections typically requires fourth-generation cephalosporins, such as cefepime (notably in cases of AmpC overexpression) or carbapenems (notably in case of extended-spectrum β-lactamase (ESBL) production).

Over the past decade, the global dissemination of carbapenemase-producing Enterobacterales (CPE) has also involved *Serratia* spp., in which all major Ambler classes of carbapenem-hydrolysing enzymes have been sporadically identified. These carbapenemases included Ambler class A enzymes (mostly KPC), Ambler class B metallo-β-lactamases (MBLs) (NDM, VIM, and IMP), and Ambler class D carbapenem-hydrolysing enzymes (OXA-48-like). Two chromosome-encoded Ambler class A carbapenemases have also been reported in *Serratia* spp., namely SME enzymes in *S. marcescens* and SFC-1 in *S. fonticola* [11]*.*

Infections caused by carbapenemase-producing *Serratia* spp. are sporadically reported, whereas documented outbreaks remain rare. From 2017 to 2024, only seven outbreaks have been reported involving different carbapenemase types: SME-4 in Argentina [12], KPC in Brazil, the United States, and South Korea [13–15], NDM in Romania [16], GES in Japan and Spain [17,18], and IMP and VIM in Egypt [19]. In most of these reports, the specific clones involved in carbapenemase production were not determined, partly because the multilocus sequence typing (MLST) scheme for *Serratia* has only been established recently [20].

The aim of this study was to elucidate the genetic diversity of carbapenemase-producing *Serratia* spp. circulating in France according to the most recent molecular taxonomy and to characterize their antimicrobial susceptibility profiles and resistance mechanisms (including intrinsic resistance to polymyxins).

## Materials and methods

### Strain collection

All *Serratia* isolates with a reduced susceptibility to at least one carbapenem (ertapenem, imipenem, or meropenem) sent to the French National Reference Center (F-NRC) for Antimicrobial Resistance from 1st January 2016 to 31st December 2024 were included (n = 193). The bacterial isolates referred to F-NRC were recovered from clinical and screening human specimens collected in French microbiology laboratories (Table S1).

### Whole-genome sequencing and bioinformatic analysis

Whole-genome sequencing was performed on all the 193 clinical isolates using the Illumina HiSeq platform (Illumina Inc., San Diego, CA, USA). Raw sequencing reads were assembled using Shovill v1.1.0 and SPAdes v3.14.0 (https://bio.tools/shovil). Multi-locus sequence typing (MLST) was conducted using the PubMLST, and resistome analysis was performed with the ResFinder database [21]. The presence of plasmids was investigated by identifying replicon sequences using PlasmidFinder v2.1.

### Phylogenetic analysis

A comprehensive phylogenetic analysis including all 193 isolates was performed. A core-genome alignment was generated, and a global maximum-likelihood phylogenetic tree was constructed (Supplementary Methods).

To contextualize our 193 isolates within a broader taxonomic and epidemiological framework, we relied on an expert taxonomic knowledge base developed by the F-NRC from complete *Serratia* genomes available in the RefSeq database (n = 2,768, 2011–2025) (Figure S1).

### SNP-based phylogeny

In addition, a single nucleotide polymorphism (SNP)-based phylogenetic analysis was performed on the five main STs identified in this study: ST-601, ST-298, ST-474, ST-477, and ST-600 (Supplementary Methods).

### Antimicrobial susceptibility testing

Antimicrobial susceptibility testing was performed by disc diffusion on Mueller-Hinton agar (Bio-Rad, Marne-la-Coquette, France) (Figure S2). Minimum inhibitory concentrations (MICs) were determined by broth microdilution using customized Sensititre microplates (Thermo Fisher Scientific) for 23 antimicrobials (Supplementary Methods).

### Conjugation experiments

Conjugation experiments were performed to assess the transferability of representative plasmids from the main sequence types identified in this study. Donor strains carrying IncX3 (ST-477), IncC (ST-601), and IncL (ST-600) plasmids were selected (Supplementary Methods).

### Assessment of lipid A modifications and potential efflux pump contribution to intrinsic resistance to polymyxins in *Serratia* spp.

To investigate the mechanisms responsible for the intrinsic resistance to polymyxin of *Serratia* spp., six isolates, including three colistin-susceptible isolates (MIC ≤ 2 mg/L) and three colistin-resistant isolates (MIC ≥ 16 mg/L) of different species (2 *S. nevei*, 1 *S. sarumanii*, and 1 *S. ureilytica*), were selected. To allow relevant comparisons, the phylogenetically closest resistant isolate was compared to the susceptible one.

Lipid A modifications were analyzed using the MALDIxin test, performed as previously described [22]. This assay was applied to detect potential structural changes in the lipid A moiety of lipopolysaccharides, particularly those involving the addition of 4-amino-4-deoxy-L-arabinose (L-Ara4N) or phosphoethanolamine.

In parallel, the potential contribution of outer membrane permeability and, more specifically, efflux pumps to colistin resistance was assessed using carbonyl cyanide m-chlorophenylhydrazone (CCCP), a protonophore that indirectly inhibits efflux activity by dissipating the proton motive force, and phenylalanine-arginine β-naphthylamide (PAβN), an outer membrane permeabilizer, according to previously described protocols [23,24] (Supplementary Methods).

## Results

### Collection overview and clinical origin

From 2016 to 2024, a total of 193 non-duplicate carbapenemase-producing *Serratia* spp. isolates were collected by the F-NRC (Table S1), representing 0.63% of all CPE received during the same period (n = 30.238). The number of isolates increased steadily over time, with only five isolates in 2016, four in 2017, and a peak of 47 in 2024 (Table S1). Half of the isolates (n = 96, 49.5%) originated from rectal screening swabs. Regarding isolates responsible for infection, they originated from urines (n = 55), blood cultures (n = 14), deep-seated infections including peritoneal fluid or soft tissue abscesses (n = 6), respiratory specimens (n = 5), and superficial wound swabs (n = 4). For 13 isolates, the clinical origin was not recorded.

**Figure 1.**
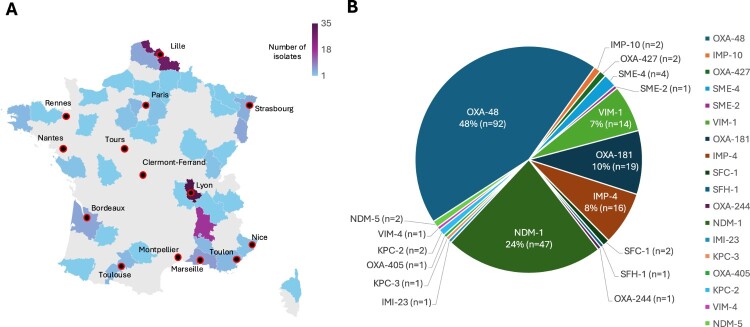
(A) Geographic distribution of the 193 carbapenemase-producing *Serratia* spp. isolates recovered in France from 2016 to 2024. Each region was shaded according to the number of isolates detected, with darker colours indicating higher counts. (B) Distribution of carbapenemases identified among the 193 *Serratia* spp. isolates collected between 2016 and 2024.

Isolates were recovered across mainland France and its overseas territories, with a notable concentration in the Lyon area (n = 56, 29%) and the Lille area (n = 36, 19%), suggesting the presence of local endemic lineages (Figure 1A). Additional isolates were identified in the Paris area (n = 17, 8.8%), the Marseille area (n = 14, 7.3%), the Strasbourg area (n = 13, 6.7%), and New Caledonia (n = 12, 6.2%), while sporadic isolates originated from multiple other regions.

### Carbapenemases and *Serratia* species distribution

Regarding carbapenemase distribution, OXA-48-like enzymes were the most prevalent (n = 108; including 88 OXA-48, 19 OXA-181, and one OXA-1167), followed by NDM-type (n = 48; 46 NDM-1 and two NDM-5), IMP-type (n = 15; three IMP-10 and 12 IMP-4), VIM-type (n = 13; 12 VIM-1 and one VIM-4), and KPC-type enzymes (n = 2). Rare carbapenemases were also identified, including two SME-4, one IMI, and one SFH-1. Two isolates co-produced multiple carbapenemases (OXA-48 + NDM-1 and OXA-48 + VIM-1) (Figure 1B).

**Figure 2.**
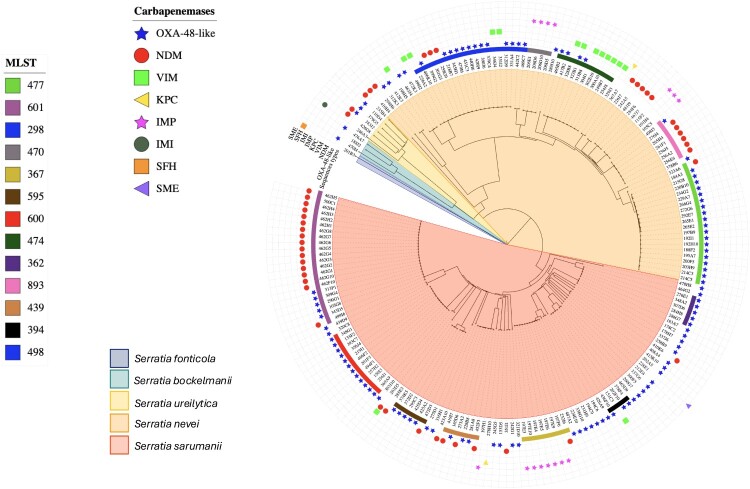
Circular phylogenetic tree of the 193 carbapenemase-producing *Serratia* isolates recovered in France from 2016 to 2024. The central tree is coloured according to the species. Carbapenemase types are indicated by distinct symbols (e.g. blue stars for OXA-48, red circles for NDM-1, etc.). The 13 most prevalent sequence types (STs) are highlighted as coloured bands surrounding the outer ring.

Species assignment was determined using average nucleotide identity (ANI) matrices of whole genomes, calculated against reference genomes representative of the *Serratia* genus. Five species were identified among the 193 isolates: *S. sarumanii* (n = 104, 54.2%), *S. nevei* (n = 79, 41.1%), *S. ureilytica* (n = 6, 3.1%), *S. bockelmannii* (n = 3, 1.6%), and *S. fonticola* (n = 1, 0.5%) (Figure 2). Interestingly, none of the isolates were identified as *Serratia marcescens.* Within the two most prevalent species, *S. sarumanii* and *S. nevei*, the distribution of carbapenemases showed distinct patterns. In *S. sarumanii*, OXA-48-like enzymes predominated (61.5%, 64/104), followed by Ambler class B (36.5%, 38/104) and Ambler class A carbapenemases (1.9%, 2/104). Conversely, in *S. nevei*, Ambler class B carbapenemases were slightly overrepresented (46.8%, 37/79, including 17 NDM, 13 VIM, and 7 IMP) compared to OXA-48-like enzymes (51.9%, 41/79).

### Positioning our collection among public genomes of *Serratia* (RefSeq)

Because the taxonomy of the *Serratia* genus remains incomplete and frequently misassigned, we integrated all complete genomes available in RefSeq (n = 2.768) with our 193 carbapenemase-producing clinical isolates (total n = 2.961). This combined dataset was used exclusively for phylogenomic and taxonomic analyses, as no antimicrobial susceptibility data were available for RefSeq genomes.

The ANI-based phylogeny (Figure S1) showed species assignments consistent with the F-NRC collection: a predominance of the *S. sarumanii* (n = 974), followed by *S. nevei* (n = 499), and the *S. marcescens/nematophila* (n = 351) which could not be resolved at the species level by ANI (Figure S1). Other *Serratia* species were represented at lower frequency. The geographic distribution of RefSeq genomes confirmed the global spread of these three dominant species. In addition, the major STs were almost exclusively confined to the two most prevalent species, herein ST-34 and ST-298 within *S. nevei*, and ST-46, ST-595, and ST-367 within *S. sarumanii*. No other species displayed recurrent high-frequency STs.

### Phylogenetic diversity and major lineages in France

On our collection, MLST analysis identified 67 distinct STs (Figure 2A). Five major STs represented 42% of our collection: ST-601 (*S. sarumanii*, n = 23), ST-298 (*S. nevei*, n = 18), ST-477 (*S. nevei*, n = 19), ST-600 (*S. sarumanii*, n = 12), and ST-474 (*S. nevei*, n = 10).

**Figure 3.**
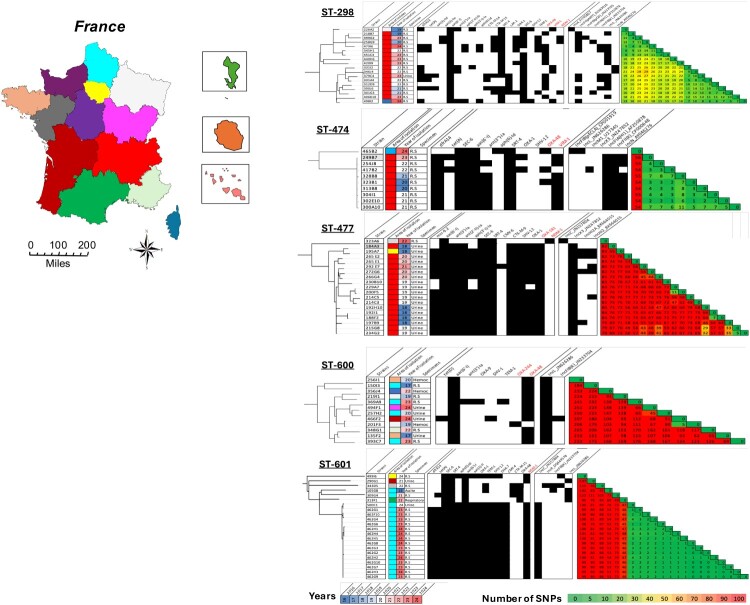
Comparative genomics of ST-298, ST-474, ST-477, ST-600 and ST-601. From left to right: core genome phylogeny (rooted on the earliest isolate within each ST), area of isolation (linked to the geographic map), year of isolation (colour gradient from blue = oldest to red = most recent), specimen type from which the *Serratia* has been isolated, presence of acquired resistance genes and plasmid replicons (black squares), and pairwise SNP distance matrix (green to red, with green indicating close relatedness and red higher divergence).

Whole-genome single nucleotide polymorphism (SNP) phylogeny was performed on each five main STs (Figure 3). The 18 *S. nevei* ST-298 isolates were predominantly recovered in the Auvergne-Rhône-Alpes region (n = 16). Despite the heterogeneous resistome, including 11 OXA-48, four VIM-type, and three NDM-type, as well as diverse plasmid backgrounds, these ST-298 *S. nevei* displayed high genomic relatedness (≤29 SNPs). The 10 *S. nevei* ST-474 isolates were all obtained from rectal swabs. Nine belonged to the same clonal cluster (≤11 SNPs) and were recovered in the Auvergne-Rhône-Alpes region. Within this cluster, seven carried VIM-1, one OXA-48, and one co-harboured both enzymes. The 19 *S. nevei* ST-477 isolates were primarily isolated from urine (n = 18). All isolates harboured a conjugative IncX3 plasmid carrying *bla*_OXA-181_, except one isolate that possessed a *bla*_NDM-1_-carrying IncC plasmid. SNP analysis revealed substantial genomic diversity (>100 SNPs), ruling out a single outbreak and suggesting the dissemination of a high-risk clone (OXA-181-producing ST-477 *S. nevei*). The 12 *S. sarumanii* ST-600 isolates were geographically scattered and derived from diverse sample types. All possessed a conjugative IncL plasmid carrying *bla*_OXA-48-like_ (11 *bla*_OXA-48_ and one *bla*_OXA-1167_), but exhibited high genetic heterogeneity (≥100 SNPs between each isolate), suggesting that *S. sarumanii* ST-600 can also be considered a high-risk clone. In contrast, the *S. sarumanii* ST-601 lineage (n = 23) provided strong evidence of a clonal expansion in one region (Hauts-de-France). Fifteen isolates harbouring a *bla*_NDM-1_-carrying conjugative IncC plasmid, recovered between 2022 and 2024 (14 from rectal swabs and one from urine), differed by ≤4 SNPs. The six remaining ST-601 isolates were more heterogeneous. They produced either OXA-48 or NDM-1 encoded by genes carried on distinct plasmid backbones and originated from different areas of France.

### Susceptibility to last resort antimicrobials

The antimicrobial susceptibility profiles of the 193 carbapenemase-producing *Serratia* spp. isolated in France were assessed by broth microdilution for 23 antimicrobials. For clarity, *Serratia* spp. isolates were divided into three groups according to the type of carbapenemase produced: Ambler class A carbapenemase producers (n = 6, including SME, SFH, IMI, and KPC), Ambler class B producers (n = 76, including NDM, VIM and IMP), and Ambler class D carbapenemase producers (n = 109, corresponding to OXA-48-like enzymes). Two isolates co-producing NDM-1 + OXA-48 and VIM-1 + OXA-48 were included within the Ambler class B group, given the predominant impact of the metallo-β-lactamases over OXA-48 on the β-lactam resistance profiles.

#### Global collection of carbapenemase-producing *Serratia* spp.

Overall, carbapenemase-producing *Serratia* spp. were highly resistant to most β-lactams, with ertapenem and imipenem showing poor activity (3% and 25% susceptibility, respectively), while meropenem retained 50% susceptibility according to the EUCAST breakpoint as updated in 2025 (Table 1). Ceftazidime and aztreonam were largely inactive (36% and 47% susceptibility), but the addition of avibactam restored full activity to aztreonam (98% susceptibility), whereas ceftazidime–avibactam remained less effective (59% susceptibility). Among novel cefepime-based regimens, cefepime–taniborbactam and cefepime–zidebactam were the most potent (>89% susceptibility).

**Table 1.** Activity of last resort β-lactam agents against *Serratia spp.* (MIC_50_, MIC_90_, susceptibility rates).

Antimicrobials	EUCAST breakpoints (mg/L)		All carbapenemase producers (n = 193)					Ambler Class B carbapenemase producers (n = 78)					Ambler Class D carbapenemase producers (n = 109)				
	S ≤	R >	MIC_50_ (mg/L)	MIC_90_ (mg/L)	%S	%I	%R	MIC_50_ (mg/L)	MIC_90_ (mg/L)	%S	%I	%R	MIC_50_ (mg/L)	MIC_90_ (mg/L)	%S	%I	%R
Ceftazidime	**1**	**4**	>16	>16	35%	2%	63%	>16	>16	0%	0%	100%	0.5	>16	59%	2%	39%
Ceftazidime–avibactam	**8**	**8**	1	>16	59%	/	41%	>16	>16	3%	/	97%	≤0.25	1	**97%**	/	3%
Cefepime	**1**	**4**	16	>16	30%	12%	58%	>16	>16	0%	13%	87%	2	>16	50%	21%	39%
Cefepime–enmetazobactam	**4**	**4**	2	>16	61%	/	39%	>16	>16	12%	/	88%	0.5	4	**94%**	/	6%
Cefepime–taniborbactam	4*	4*	0.5	4	**94%**	/	6%	1	2	87%	/	13%	≤0.25	2	**99%**	/	1%
Cefepime–zidebactam	4*	4*	0.5	8	89%	/	11%	2	4	73%	/	27%	≤0.25	1	**99%**	/	1%
Aztreonam	**1**	**4**	4	>16	47%	3%	50%	>16	>16	35%	6%	59%	≤0.25	>16	57%	3%	40%
Aztreonam–avibactam	**4**	**4**	≤0.25	0.5	**98%**	/	2%	≤0.25	≤0.25	**100%**	/	0%	≤0.25	0.5	**98%**	/	2%
Cefiderocol	**2**	**2**	0.12	4	82%	/	18%	0.5	4	72%	/	28%	≤0.12	2	**91%**	/	9%
Ertapenem	**0.5**	**0.5**	8	>16	3%	/	97%	8	>16	0%	/	100%	4	>16	0%	0%	100%
Imipenem	**2**	**4**	8	>16	19%	25%	56%	8	>16	13%	14%	73%	4	16	23%	34%	43%
Imipenem-relebactam	**2**	**2**	4	>16	25%	/	75%	16	>16	13%	/	88%	4	16	32%	/	68%
Meropenem	**2**	**8**	2	16	50%	34%	16%	8	>16	55%	24%	22%	2	16	57%	31%	12%
Meropenem-vaborbactam	**8**	**8**	2	16	87%	/	13%	4	>16	83%	/	17%	2	16	89%	0%	11%
Gentamicin	**2**	**2**	16	>16	45%	/	55%	>16	>16	17%	/	83%	≤0.5	>16	66%	/	34%
Amikacin	**8**	**8**	4	>16	73%	/	27%	16	>16	45%	/	55%	4	8	**92%**	/	8%
Ciprofloxacin	**0.25**	**0.5**	1	>2	43%	6%	51%	1	>2	26%	10%	64%	0.25	>2	55%	3%	42%
Levofloxacin	**0.5**	**1**	0.5	>2	68%	9%	23%	0.5	>2	72%	10%	18%	0.25	>2	65%	7%	28%
Tigecyclin	**–**	**–**	1	2	–	–	–	1	2	–	–	–	1	2	–	–	–
Eravacyclin	**–**	**–**	1	2	–	–	–	1	2	–	–	–	1	2	–	–	–
Colistin	**2**	**2**	>64	>64	2%	/	98%	>64	>64	0%	/	100%	>64	>64	3%	/	97%

“*”, cefepime breakpoints were used; “–” no EUCAST breakpoint; “/”, no I categorization. The percentages of susceptibility ≥90% were bolded.

#### Ambler class A carbapenemase producers

Six Ambler class A carbapenemase producers were identified, including SME-4 (*S. bockelmannii* and *S. sarumanii*), SFH-1/SFC-2 (*S. fonticola*), IMI-like (*S. ureilytica*), and two KPC-producers (one KPC-2-producing *S. nevei* and one KPC-3-producing *S. sarumanii*) (Table S1).

SME-4 and IMI-like producers exhibited high-level resistance to carbapenems and aztreonam, but remained susceptible to ceftazidime, cefepime, and to all β-lactam/β-lactamase inhibitor combinations (Figure S2-A-B-D, Table S1).

The *S. fonticola* isolate, co-producing SFH-1 (class B) and SFC-2 (class A), displayed susceptibility to ceftazidime, cefepime, aztreonam, ertapenem, and meropenem. Imipenem was the only carbapenem with a high MIC (>16 mg/L) not decreased by addition of relebactam (Figure S2-C, Table S1).

The two KPC producers were susceptible to all news β-lactam/β-lactamase inhibitor combinations (Figure S2-E, Table S1).

#### Metallo-β-lactamase (MBL) producers

**Figure 4.**
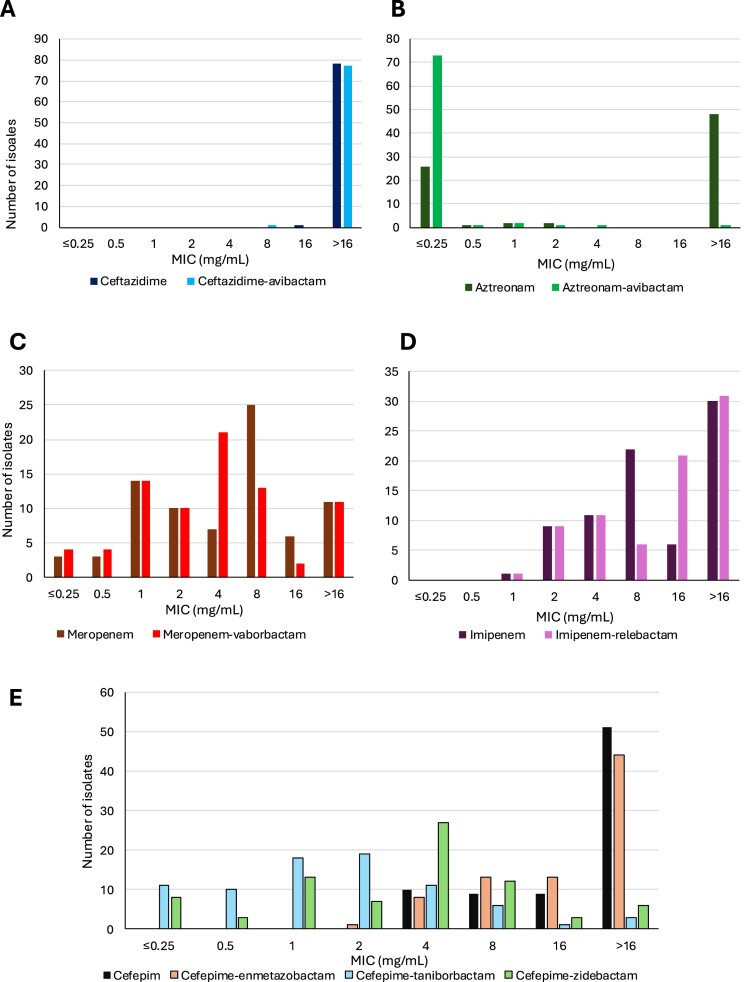
Minimal inhibitory concentration distribution of β-lactams/β-lactamases inhibitors MICs for metallo-β-lactamase– producing *Serratia* spp. MIC distributions are shown for ceftazidime ± avibactam (panel A), aztreonam ± avibactam (panel B), meropenem ± vaborbactam (panel C), imipenem ± relebactam (panel D), and cefepime with or without β-lactamase inhibitors (enmetazobactam, taniborbactam, zidebactam) (panel E).

Among the 78 MBL producers (including the 2 co-producers OXA-48 + MBL), the highest susceptibility rate was obtained for aztreonam–avibactam (100%), followed by cefepime–taniborbactam (87%), cefepime–zidebactam (83%), and cefiderocol (82%) (Figure 4, Table 1). Cefepime displayed no significant intrinsic activity (MIC_50_ and MIC_90_ > 16 mg/L), but combinations with zidebactam of taniborbactam improved its efficacy. Indeed, cefepime–zidebactam and cefepime–taniborbactam achieved MIC_50_ and MIC_90_ values of 2 and 4 mg/L, and 1 and 2 mg/L, respectively. As expected, the addition of enmetazobactam did not restore cefepime susceptibility. Of note, susceptibility to cefepime–taniborbactam, cefepime–zidebactam and cefiderocol varied depending on the MBL enzyme (Table S1). NDM producers (n = 48) remained susceptible at 93.8% (45/48), 70.1% (34/48) and 60.4% (29/48) to cefepime–taniborbactam, cefepime–zidebactam and cefiderocol. For VIM producers (n = 13), these susceptibility rates were of 100% (13/13), 84.6% (11/13), and 92.3% (12/13). As expected, the addition of taniborbactam was ineffective in inhibiting IMP enzymes, leading to identical susceptibility rates (46.7%, 7/15) for cefepime alone and cefepime–taniborbactam. Cefepime–zidebactam and cefiderocol were more effective on IMP producers with susceptibility rates of 80% (12/15) and 93.3% (14/15), respectively.

The most effective non-β-lactam antibiotics were levofloxacin (72% susceptibility) and amikacin (45% susceptibility).

#### Ambler class D carbapenemase producers (OXA-48-like)

**Figure 5.**
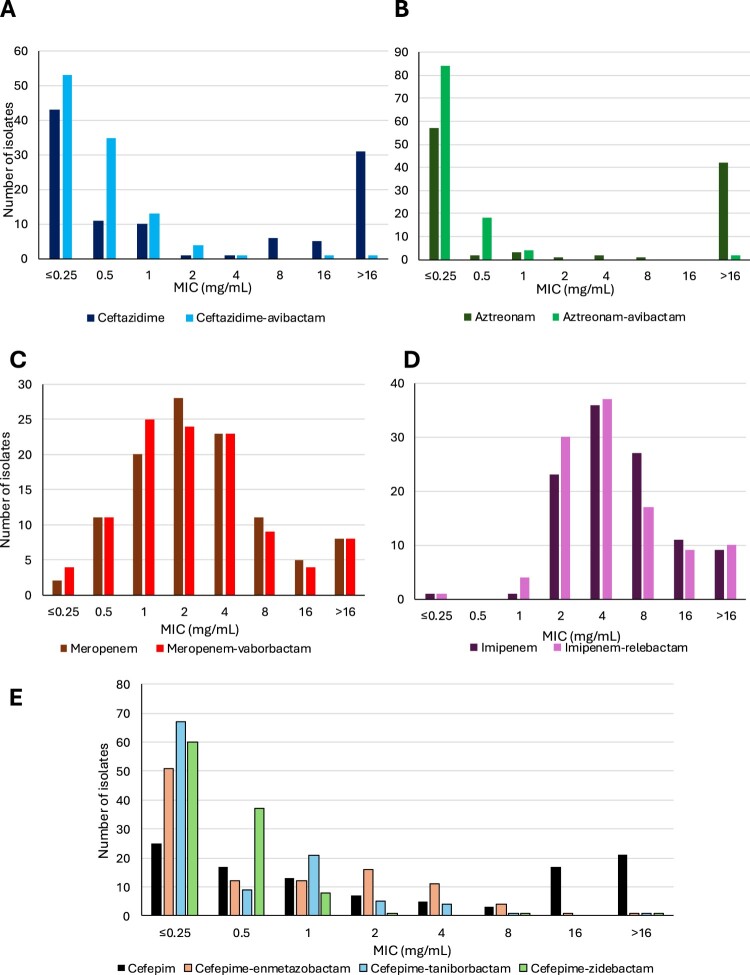
Minimal inhibitory concentration distributions of β-lactams/β-lactamases inhibitors MICs for OXA-48-like–producing *Serratia* spp. MIC distributions are shown for ceftazidime ± avibactam (panel A), aztreonam ± avibactam (panel B), meropenem ± vaborbactam (panel C), imipenem ± relebactam (panel D), and cefepime with or without β-lactamase inhibitors (enmetazobactam, taniborbactam, zidebactam) (panel E).

Overall, the OXA-48-like producers (n = 108) were more susceptible than MBL producers (Figure 5, Table 1). Seven antimicrobials displayed susceptibility rates over 95%, including ceftazidime–avibactam, cefepime–enmetazobactam, cefepime–taniborbactam, cefepime–zidebactam, aztreonam–avibactam, cefiderocol, and amikacin. For β-lactam/β-lactamase inhibitor association, the addition of the inhibitor markedly restored the efficacy of the associated β-lactam (ceftazidime, cefepime, or aztreonam), which was not hydrolyzed by the OXA-48-like enzyme itself but by associated β-lactamases (e.g. ESBL).

### Deciphering intrinsic resistance to polymyxins

Despite *Serratia* spp. being known to be intrinsically resistant to polymyxins, the resistance mechanism remains poorly understood. Interestingly, among the 193 carbapenemase-producing *Serratia* of our collection, four isolates (2.1%) remained susceptible to colistin (MIC ≤2 mg/L). These included two *S. sarumanii*, one *S. ureilytica*, and one *S. nevei* (Figure S3). As expected, the remaining 189 isolates displayed high-level resistance to colistin, with MIC_90_ values ≥32 mg/L, confirming the intrinsic resistance of *Serratia* spp. to this molecule, often used as a last resort antimicrobial for the treatment of infections caused by carbapenemase-producing Enterobacterales.

To investigate whether lipid A modifications were responsible for polymyxin resistance as recently demonstrated for another intrinsically resistant organism, *Morganella* spp. [25], the MALDIxin test was performed on phylogenetically close pairs (susceptible vs. resistant). Contrary to what was previously observed in *Morganella* spp. [25], no differences in lipid A spectra were obtained between susceptible and resistant *Serratia* spp. isolates, suggesting that intrinsic polymyxin resistance in this genus is not driven by specific lipid A modifications. Thus, the role of outer membrane permeability in colistin resistance was investigated using carbonyl cyanide m-chlorophenylhydrazone (CCCP), a protonophore that dissipates the proton motive force required for the activity of RND-type efflux systems, and phenylalanine-arginine β-naphthylamide (PAβN), an outer membrane permeabilizer commonly used as an efflux pump inhibitor [26]. The addition of CCCP significantly restored colistin susceptibility for all colistin-resistant isolates, with a significant increase in the inhibition zone diameter from 6 mm to 23, 19, and 17 mm for *S. nevei* 329J3, *S. sarumanii* 348A2, and *S. ureilytica* 235B4, respectively (Figure S3). Of note, the intrinsic activity of CCCP alone was ruled out since the inhibition zone diameter of colistin-susceptible *Serratia* spp. (*S. nevei* 367A7, *S. sarumanii* 276E1, and *S. ureilytica* 110D4) was not modified by the addition of CCCP. In contrast, PAβN did not restore colistin susceptibility in any of the tested isolates (Table S2).

## Discussion

Since *Serratia* spp. have been comparatively underexplored, this study was designed to provide an integrated and comprehensive characterization of the genus, combining updated taxonomic insight, population structure, and resistance mechanisms (β-lactamines and polymixines).

Among carbapenemase-producing *Serratia* spp. isolates referred to the F-NRC from 2016 to 2024, the most frequent species were *S. sarumanii* (54%) and *S. nevei* (41%), followed by sporadic isolates of *S. ureilytica*, *S. bockelmannii*, and *S. fonticola*. Our data also highlighted the absence of *S. marcescens sensu stricto* in multidrug-resistant *Serratia* spp. in France, despite its presence being reported in Asia [27]. The absence of *S. marcescens*, historically considered the reference species and commonly identified by MALDI-TOF mass spectrometry, underscores the long-standing under-recognition of other *Serratia* species in clinical microbiology.

Worldwide, *S. sarumanii* emerged as the main circulating species across continents, followed by *S. nevei*, particularly in Europe and North America. Regional clusters were also evidenced, with *S. marcescens/nematodiphila* mostly confined to Asia, *S. bockelmannii* enriched in Europe, and *S. ureilytica* in Europe and North America (Figure S1). These findings underscore the need to refine *Serratia* taxonomy, as exemplified by the fact that *S. marcescens* and *S. nematodiphila* appear extremely close and may ultimately represent a single species best separated into subspecies, as suggested by Williams *et al.* [28]. Despite being the most prevalent species in our collection, Williams *et al.* did not report any *S. sarumanii* or *S. nevei* isolates in their collection [28]. This discrepancy may partly reflect differences in the sources of isolates, as Williams *et al.'s* collection included strains from diverse environments, whereas our carbapenemase-producing *Serratia* isolates were exclusively of human origin.

Interestingly, in our collection *S. nevei* isolates harboured a higher proportion of MBLs compared with *S. sarumanii*. To ensure that this difference was not driven by clonal expansion, we performed SNP-based phylogenetic analyses. Among the 67 distinct STs, we identified major lineages corresponding to ST-34 and ST-298 within *S. nevei*, and ST-46, ST-595, and ST-367 within *S. sarumanii*. Notably, ST-474 and ST-601 appeared to be associated with local outbreak events. In particular, NDM-1-producing *S. sarumanii* ST-601 was responsible for a large outbreak in a haematology ward in northern France (data not shown), with a maximum pairwise SNP distance of 4, suggesting recent transmission. Of note, this lineage has already been reported as a major NDM-1-carrying ST among *Serratia* spp. [27], underscoring its epidemic potential. Similarly, ST-474 appeared to correspond to a local clonal dissemination in the Auvergne-Rhône-Alpes region, with a maximum pairwise SNP distance of 11 between isolates. Although no validated SNP threshold exists for *Serratia* spp., these low SNP distances are consistent with potential transmission events.

Several clones showed distinct epidemiological behaviours. ST-298, ST-474, and ST-477 predominated in Auvergne-Rhône-Alpes, a recognized carbapenemase hotspot in France [28,29], displayed limited chromosomal variation (<25 SNPs) but heterogeneous plasmids and carbapenemases encoding genes (*bla*_OXA-48_, *bla*_NDM-1_, *bla*_VIM-1_), whereas ST-477 showed higher genomic diversity (>100 SNPs) despite a conserved resistome, mostly *bla*_OXA-181_-carrying incX3 plasmids [30], which are known to be highly conjugative [31]. Notably, 18 of 19 ST-477 isolates originated from urinary samples, suggesting a possible urinary tropism of this particular ST. Finally, ST-600, scattered across France, was mainly associated with bloodstream and urinary tract infections, and consistently carried IncL plasmid harbouring *bla*_OXA-48-like_, known for their high dissemination potential [32].

Beyond taxonomy, our findings shed light on resistance mechanisms. As previously described, S*erratia* spp. intrinsically harbour an aminoglycoside resistance gene *aac(6’)-Ib* [30] and display intrinsic resistance to polymyxins. This intrinsic resistance to polymyxins has been reported to be caused by lipid A modifications (phosphoethanolamine addition) mediated by EptB, a phosphoethanolamine transferase [31]. Interestingly, four isolates were colistin-susceptible in our collection, with no specific species association. The MALDIxin analysis revealed that no lipid A modification was responsible for colistin resistance. CCCP assays indicated that proton motive force (PMF)-dependent mechanisms may contribute to intrinsic resistance, as susceptibility was restored upon PMF disruption. However, this effect is unlikely to reflect a direct involvement of RND-type efflux pumps such as SdeAB-HasF, as previously suggested [23], since PAβN had no impact on colistin susceptibility. These findings rather suggest that PMF disruption alters outer membrane electrostatic properties, thereby affecting colistin–lipid A interactions.

Regarding acquired resistance mechanisms and particularly carbapenemase, OXA-48-like enzymes represented the most prevalent carbapenemases, followed by NDM, IMP, and VIM metallo-β-lactamases. The predominance of IMP over VIM, although uncommon among Enterobacterales in France mainland, mostly reflected localized outbreaks in New Caledonia and La Réunion Islands. Overall, this distribution mirrors the national epidemiology of carbapenemases in France [33] with no species-specific associations.

Regarding antimicrobial options available for the treatment of infections caused by carbapenemase-producing *Serratia* spp., aztreonam–avibactam showed the most consistent activity. Overall, cefepime–taniborbactam displayed higher activity than cefepime–zidebactam for MBL producers, except IPM-producing isolates, as previously described [34,35]. In particular, IMP-10-producers remained resistant to all tested cefepime-inhibitors combinations, whereas those producing IMP-4 retained partial susceptibility to cefepime–zidebactam. These findings highlight the need to evaluate next-generation inhibitors such as xeruborbactam, which was reported to retain inhibition activity against most IMP-type enzymes [34]. The fact that zidebactam has been reported to possess an intrinsic antibiotic activity *via* PBP2 binding [36] but shows no effect alone against *Serratia* spp. [36] might indicate a species-PBP2 modification reducing its activity in *Serratia*, similar to what has been described in the tribe Proteae (including in *Morganella* and *Proteus* spp. and *Providencia*) [25,37]. Further investigations are warranted to confirm this hypothesis. However, to support this hypothesis, mecillinam, which also targets PBP2, has been shown to display poor activity against carbapenemase-producing *Serratia* [10]

Regarding SME-4 and IMI-23 Ambler class A carbapenemases, they conferred resistance to carbapenems mostly (particularly imipenem), while susceptibility was observed for the other β-lactams. As expected, carbapenem resistance was efficiently counteracted by Ambler class A inhibitors such as relebactam and vaborbactam [38]. By contrast, the co-production of SFC-1 and SFH-1 resulted in a refractory phenotype, combining an Ambler class A enzyme (SFC-1) with a metallo-β-lactamase (SFH-1). Notably, IMI-23 and SFC-1/SFH-1 had so far only been detected in environmental *S. fonticola* in Japan [39], and remained absent from clinical settings before our description (here, the SFC-1/SFH-1 has been isolated from a urine sample).

To date, this study provides the most comprehensive characterization of carbapenemase-producing *Serratia* spp. in Europe. We demonstrated that most of *Serratia* spp. isolates previously identified as *S. marcescens* finally belonged to *S. sarumanii* and *S. nevei*. Our results highlighted *S. sarumanii* ST-600 as a potential high-risk clone associated with severe infections, and *S. nevei* ST-477 as a non-clonal lineage disseminating an *bla*_OXA-181_-carrying incX3 plasmid. We also demonstrated that cefepime–taniborbactam consistently outperformed cefepime–zidebactam, suggesting possible species-specific PBP2 modifications as previously described for *Morganella* and *Proteus*. In addition, our findings revealed that colistin resistance in *Serratia* is likely caused by efflux pump activity rather than lipid A modification, contrary to what has been described for other intrinsically resistant Enterobacterales such as *Morganella* spp. [25]. Rather than supporting a direct role of efflux pumps, our results point towards a proton motive force-dependent mechanism, possibly involving alterations of outer membrane electrostatic properties. Compounds disrupting the proton gradient, as previously explored in *Acinetobacter* spp*.* [40]*,* may therefore help restore colistin susceptibility, which could be of particular interest for carbapenemase-producing *Serratia* isolates with limited therapeutic options. Finally, by placing our data in a global context, we showed that *S. sarumanii* and *S. nevei* dominate worldwide, reshaping the current understanding of the *Serratia* clinical landscape. These results strongly suggest that the 2022 *Serratia* taxonomy [28] is no longer adequate, and a major taxonomic revision of the genus is urgently needed.

Our study nonetheless has some limitations. No validated SNP threshold is currently available to define clonality in *Serratia* spp., making it difficult to establish robust criteria for transmission inference based solely on genomic distances. The use of short-read Illumina sequencing limited plasmid reconstruction and the analysis of carbapenemase gene mobilization. Moreover, although our results suggest the contribution of efflux pump(s) to polymyxin resistance, complementary assays will be required to definitively identify the efflux system(s) involved.

## Supplementary Material

Supplementary data_revised.docx

Graphical abstract.pdf
