# Supplementary material for: Spread of carbapenemase-producing Serratia spp. in France from 2016 to 2024: a comparative genomic study
Source: Emerg Microbes Infect. 2026 May 7;15(1):2671515. doi: 10.1080/22221751.2026.2671515 (PMC13224694; doi:10.1080/22221751.2026.2671515)
Supplement: Supplementary data_revised.docx [file TEMI_A_2671515_SM3330.docx]

**Spread of carbapenemase-producing *Serratia* spp. in France from 2016 to 2024: a comparative genomic study**

Inès REZZOUG^1,2,3,4^, Sandrine BERNABEU^1,2,4^, Kenza OUACEL^1^, Aurelien BIRER ^5,6^, Agnès B. JOUSSET^1,2,3,4^, Gerald LAROUY-MAUMUS^7^, Rémy A. BONNIN^2,3,4^, Cécile EMERAUD^1,2,3,4^, Laurent DORTET^1,2,3,4^

**SUPPLEMENTARY DATA**

**Supplementary Methods**

**Figure S1.** **Phylogenomic analysis and taxonomic assignment of *Serratia* isolates.**
ANI-based phylogenetic tree including *Serratia* isolates from the French National Reference Center (CNR-KB, blue) and the RefSeq database (yellow). Branch colors indicate species or species complexes, including *S. marcescens* complex, *S. liquefaciens* complex, *S. fonticola* complex, *S. rubidaea* complex, *S. ureilytica*, *S. entomophila*, *S. quinivorans*, *S. symbiotica*, *S. aquatilis*, *S. grimesii*, and others. Major sequence types (STs) are indicated in the inner ring, while the outer ring denotes isolate origin (CNR-KB vs RefSeq). The world map (top right) shows the global distribution of isolates according to species assignment. The ANI heatmap (bottom right) confirms genomic clustering into distinct *Serratia* species/complexes, supporting taxonomic delineation across clinical (CNR-KB) and environmental (RefSeq) isolates.

**Figure S2.** Antimicrobial susceptibility testing of class A carbapenemases including **(A)** SME-4-producing *Serratia bockelmannii* (188J2), **(B)** SME-4-producing *Serratia sarrumani*, SME-4 carbapenemase producers (228 E1), **(C)** SFC-2 & SFH-1-producing *Serratia fonticola* (101B3), **(D)** IMI-23-producing *Serratia ureilytica* (246A3), **(E)** KPC-2-producing *Serratia nevei* (367A7). Amoxicillin (AX), amoxicillin-clavulanic acid (AMC), ticarcillin (TIC), ticarcillin-clavulanic acid (TIM), piperacillin (PRL), piperacillin-tazobactam (TZP), cefoxitin (FOX), cefotaxime (CTX), ceftazidime (CAZ), cefepime (FEP), cefiderocol (CID), aztreonam (ATM), mecillinam (MEC), temocillin (TEM), imipenem (IPM), meropenem (MEM), ertapenem (ETP), ceftazidime-avibactam (CTV), amikacin (AK), gentamicin (CN), eravacycline (ERA), tigecycline (TGC), fosfomycin (FF), chloramphenicol (C), sulfamethoxazole/trimethoprim (SXT), nitrofurantoin (F), nalidixic acid (NA), pefloxacin (PEF), levofloxacin (LEV), ciprofloxacin (CIP) and colistin (CT).

**Figure S3.** Colistin susceptibility testing of carbapenemase-producing *Serratia* spp. isolates using disc diffusion. Representative strains included *S. nevei* (367A7, 329J3), *S. sarumanii* (276E1, 348A2), and *S. ureilytica* (110D4, 235B4). For each strain, inhibition of the oxidative phosphorylation was assessed using carbonyl cyanide m-chlorophenyl hydrazone (CCCP), with dimethyl sulfoxide (DMSO) used as the solvent control.

**Table S1**. **Characteristics of the 193 carbapenemase-producing Serratia spp. isolates included in this study.** Table providing detailed metadata for all isolates analyzed, including strain identifier, postal code, departement, date of isolation, and clinical specimen. Species assignment and MLST type are reported alongside the carbapenemase and its Ambler class. Minimum inhibitory concentrations (MICs) for all antibiotics tested in this study are also summarized.

**Table S2: Colistin MICs in the presence or absence of PAβN**. This table presents MIC values for six isolates, including two S. nevei, two S. sarumanii, and two S. ureilytica, selected based on close phylogenetic relatedness. For each species, one intrinsically resistant isolate and one intrinsically susceptible isolate were included.

**Supplementary Methods**

***Bacterial identification***

The bacterial identification of all 193 Serratia spp. isolates was verified by MALDI-TOF mass spectrometry (Biotyper, Bruker Daltonics).

***Carbapenemase detection***

Carbapenemase detection was performed using Carba NP test as previously described, followed by an immunochromatographic detection of the carbapenemase enzyme using NG-Carba5 test (NG Biotech, Guipry, France) [1].

***Minimum inhibitory concentrations (MICs)***

Minimum inhibitory concentrations (MICs) were determined by broth microdilution using a customized Sensititre microplates (Thermo Fisher Scientific) for the following antibiotics: aztreonam, colistin, imipenem, cefepime, cefiderocol, meropenem, meropenem-vaborbactam, imipenem-relebactam, ceftazidime-avibactam, ceftazidime, aztreonam-avibactam, ertapenem, temocillin, cefepime-enmetazobactam, cefepime-taniborbactam, cefepime-zidebactam, gentamicin, amikacin, ciprofloxacin, levofloxacin, tigecycline and eravacycine. According to EUCAST recommendations, the concentration of β-lactamase inhibitors was fixed at 4 mg/L, except for enmetazobactam which was tested at 8 mg/L fixed concentration, and cefepime-zidebactam for which a 1:1 ratio was used. Results were interpreted according to EUCAST clinical breakpoints version 15.0 (2025). As no EUCAST breakpoints were available for cefepime-zidebactam and cefepime-taniborbactam, those of cefepime alone cefepime-enmetazobactam (≤4 mg/L / >4 mg/L) were applied.

***Phylogenetic analysis***

A comprehensive phylogenetic analysis including all 193 isolates was performed. A core-genome alignment was generated, and a global maximum-likelihood phylogenetic tree was constructed (Supplementary Methods). The resulting tree was visualized and annotated using iTOL v6.5.2 (European Molecular Biology Laboratory).

Given the historically inconsistent and outdated taxonomy of the Serratia genus, species identification for each isolate was verified according to the updated nomenclature available on bacterio.net (LPSN, List of Prokaryotic names with Standing in Nomenclature, <https://www.bacterio.net/>). To support this classification, a curated set of 25 Serratia genomes with validly published names was selected from LPSN and used as taxonomic references.Average Nucleotide Identity (ANI) analysis was performed using FastANI (v1.34) <https://proksee.ca/tools/fastani> . Pairwise ANI values were calculated between these taxonomically validated reference genomes and all isolates, and a threshold of ≥95% ANI was applied for species delineation <https://pubmed.ncbi.nlm.nih.gov/30504855/> . Species assignments were then integrated into the phylogenetic framework.

To provide a broader taxonomic and epidemiological context, these reference genomes, together with 2,768 publicly available Serratia genomes retrieved from the RefSeq database, were included in the phylogenetic analysis. All genomes (reference, RefSeq, and study isolates) were integrated into the tree and visualized using iTOL. Associated metadata, including geographical origin, were extracted from RefSeq records for downstream analyses.

***SNP-based phylogeny***

For each ST, the earliest available isolate was selected as the reference genome: strain 105G8 for ST-601, 218B7 for ST-298, 249B7 for ST-474, 184A3 for ST-477, and 135F2 for ST-600. Sequencing reads from each group were mapped to their corresponding reference genome using Snippy v4.6.0, and SNPs matrix were constructed to infer phylogenetic relationships. ST-specific trees were constructed and visualized using iTOL v6.5.2.

***Conjugation experiments***

Conjugation was carried out using a filter mating method with azide-resistant *Escherichia coli* J53 as the recipient strain. Transconjugants were selected on selective media containing ticarcillin (50 mg/L) and sodium azide (50 mg/L). The presence of the transferred plasmids in transconjugants was confirmed using the NG-Test Carba 5 assay (NG Biotech, Guipry, France) [1], targeting the corresponding carbapenemase enzymes.

***Assessment of potential efflux pump contribution to intrinsic resistance to polymyxins in*** Serratia ***spp.***

Disk diffusion assays were performed for both colistin-susceptible and colistin-resistant isolates on Mueller–Hinton agar (Bio-Rad) under three conditions: (i) agar alone, (ii) agar supplemented with 0.2% dimethyl sulfoxide (DMSO), and (iii) agar supplemented with 0.2% DMSO and 10 mg/L carbonyl cyanide m-chlorophenyl hydrazone (CCCP). A 50-µg colistin disk was used to assess the inhibition zone diameter.

In addition, the involvement of efflux pumps was further investigated using phenylalanine-arginine β-naphthylamide (PAβN). Bacterial growth was assessed in the presence of increasing concentrations of colistin combined with a fixed concentration of PAβN (30 µg/mL). Growth inhibition was evaluated to determine the potential contribution of efflux mechanisms to colistin resistance.

[1] Dortet L, Bréchard L, Cuzon G, et al. Strategy for rapid detection of carbapenemase-producing Enterobacteriaceae. *Antimicrob Agents Chemother* 2014; 58: 2441–2445.

**Figure S1.** **Phylogenomic analysis and taxonomic assignment of *Serratia* isolates.**

ANI-based phylogenetic tree including *Serratia* isolates from the French National Reference Center (CNR-KB, blue) and the RefSeq database (yellow). Branch colors indicate species or species complexes, including *S. marcescens* complex, *S. liquefaciens* complex, *S. fonticola* complex, *S. rubidaea* complex, *S. ureilytica*, *S. entomophila*, *S. quinivorans*, *S. symbiotica*, *S. aquatilis*, *S. grimesii*, and others. Major sequence types (STs) are indicated in the inner ring, while the outer ring denotes isolate origin (CNR-KB vs RefSeq). The world map (top right) shows the global distribution of isolates according to species assignment. The ANI heatmap (bottom right) confirms genomic clustering into distinct *Serratia* species/complexes, supporting taxonomic delineation across clinical (CNR-KB) and environmental (RefSeq) isolates.

To contextualize the 193 isolates analysed in this study within a broader taxonomic and epidemiological framework, we relied on an expert taxonomic knowledge base developed by the French National Reference Center (F-NRC) from complete Serratia genomes available in the RefSeq database (n=2,768, 2011–2025). This curated database, routinely used by the F-NRC for species-level identification of newly sequenced isolates, served as a reference for our taxonomic assignments. Several isolates from our collection clustered within unpublished taxonomic groups present in this F-NRC database. To further characterize these unclassified lineages and confirm their taxonomic validity, average nucleotide identity (ANI) analyses were performed alongside the global Serratia phylogeny. Species boundaries were defined using an ANI threshold of ≥ 95 %, supporting the delineation of these novel lineages within the genus and providing an updated overview of Serratia diversity and evolution.

**Figure S2.** Antimicrobial susceptibility testing of class A carbapenemases including **(A)** SME-4-producing *Serratia bockelmannii* (188J2), **(B)** SME-4-producing *Serratia sarrumani*, SME-4 carbapenemase producers (228 E1), **(C)** SFC-2 & SFH-1-producing *Serratia fonticola* (101B3), **(D)** IMI-23-producing *Serratia ureilytica* (246A3), **(E)** KPC-2-producing *Serratia nevei* (367A7). Amoxicillin (AX), amoxicillin-clavulanic acid (AMC), ticarcillin (TIC), ticarcillin-clavulanic acid (TIM), piperacillin (PRL), piperacillin-tazobactam (TZP), cefoxitin (FOX), cefotaxime (CTX), ceftazidime (CAZ), cefepime (FEP), cefiderocol (CID), aztreonam (ATM), mecillinam (MEC), temocillin (TEM), imipenem (IPM), meropenem (MEM), ertapenem (ETP), ceftazidime-avibactam (CTV), amikacin (AK), gentamicin (CN), eravacycline (ERA), tigecycline (TGC), fosfomycin (FF), chloramphenicol (C), sulfamethoxazole/trimethoprim (SXT), nitrofurantoin (F), nalidixic acid (NA), pefloxacin (PEF), levofloxacin (LEV), ciprofloxacin (CIP) and colistin (CT).

**Figure S3.** Colistin susceptibility testing of carbapenemase-producing *Serratia* spp. isolates using disc diffusion. Representative strains included *S. nevei* (367A7, 329J3), *S. sarumanii* (276E1, 348A2), and *S. ureilytica* (110D4, 235B4). For each strain, inhibition of the oxidative phosphorylation was assessed using carbonyl cyanide m-chlorophenyl hydrazone (CCCP), with dimethyl sulfoxide (DMSO) used as the solvent control.

**Table S1**: **Characteristics of the 193 carbapenemase-producing Serratia spp. isolates included in this study.** Table providing detailed metadata for all isolates analyzed, including strain identifier, postal code, departement, date of isolation, and clinical specimen. Species assignment and MLST type are reported alongside the carbapenemase and its Ambler class. Minimum inhibitory concentrations (MICs) for all antibiotics tested in this study are also summarized.

**Table S2: Colistin MICs in the presence or absence of PAβN**. This table presents MIC values for six isolates, including two S. nevei, two S. sarumanii, and two S. ureilytica, selected based on close phylogenetic relatedness. For each species, one intrinsically resistant isolate and one intrinsically susceptible isolate were included.

|  | **Strains** | **MIC colistin** | **MIC colistin + PAβN** |
| --- | --- | --- | --- |
| *S. nevei* | 367A7 | 2 mg/mL | 2 mg/mL |
| *S. nevei* | 329J3 | >64 mg/mL | >64 mg/mL |
| *S. sarumanii* | 276E1 | 1 mg/mL | 1 mg/mL |
| *S. sarumanii* | 348A2 | >64 mg/mL | >64 mg/mL |
| *S. ureilytica* | 110D4 | 1 mg/mL | 1 mg/mL |
| *S. ureilytica* | 235B4 | >64 mg/mL | >64 mg/mL |
