# Supplementary material for: Spread of carbapenemase-producing Serratia spp. in France from 2016 to 2024: a comparative genomic study
Source: Emerg Microbes Infect. 2026 May 7;15(1):2671515. doi: 10.1080/22221751.2026.2671515 (PMC13224694; doi:10.1080/22221751.2026.2671515)

193 carbapenemase-producing *Serratia* spp. (France, 2016 to 2024)

Geographic distribution

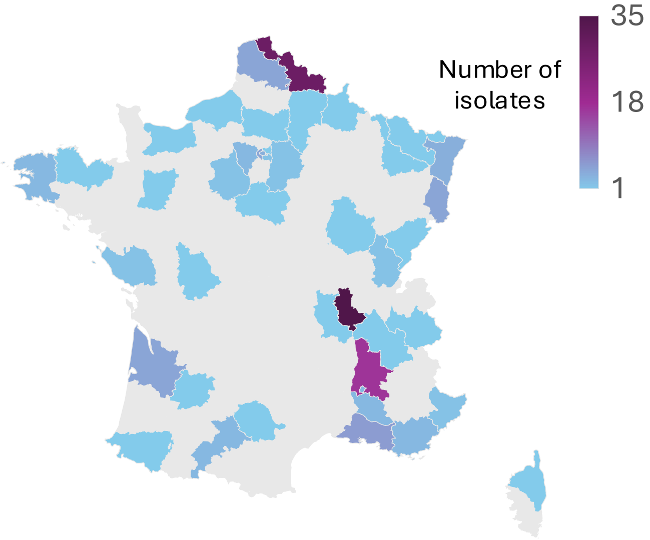

Carbapenemases

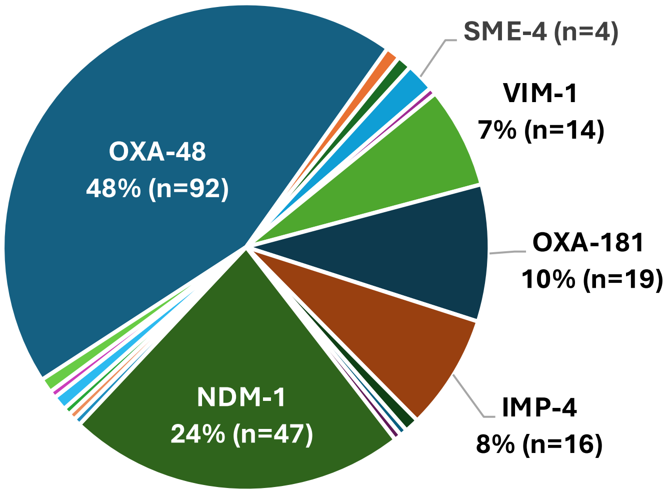

Antimicrobial susceptibility

Antimicrobials	All carbapenemase (n=193)	Ambler Class B carbapenemase (n=78)	Ambler Class D carbapenemase (n=109)
	%S + I	%S + I	%S + I
Ceftazidime	37%	0%	61%
Ceftazidime-avibactam	59%	3%	97%
Cefepime	42%	13%	71%
Cefepime-enmetazobactam	61%	12%	94%
Cefepime-taniborbactam	94%	87%	99%
Cefepime-zidebactam	89%	73%	99%
Aztreonam	50%	41%	60%
Aztreonam-avibactam	98%	100%	98%
Cefiderocol	82%	72%	91%
Ertapenem	3%	0%	0%
Imipenem	44%	27%	57%
Imipenem-relebactam	25%	13%	32%
Meropenem	84%	79%	88%
Meropenem-vaborbactam	87%	83%	89%
Gentamicin	45%	17%	66%
Amikacin	73%	45%	92%
Ciprofloxacin	49%	36%	58%
Levofloxacin	77%	82%	71%
Colistin	2%	0%	3%

0%	10%	20%	30%	40%	50%	60%	70%	80%	90%	100%

Molecular epidemiology

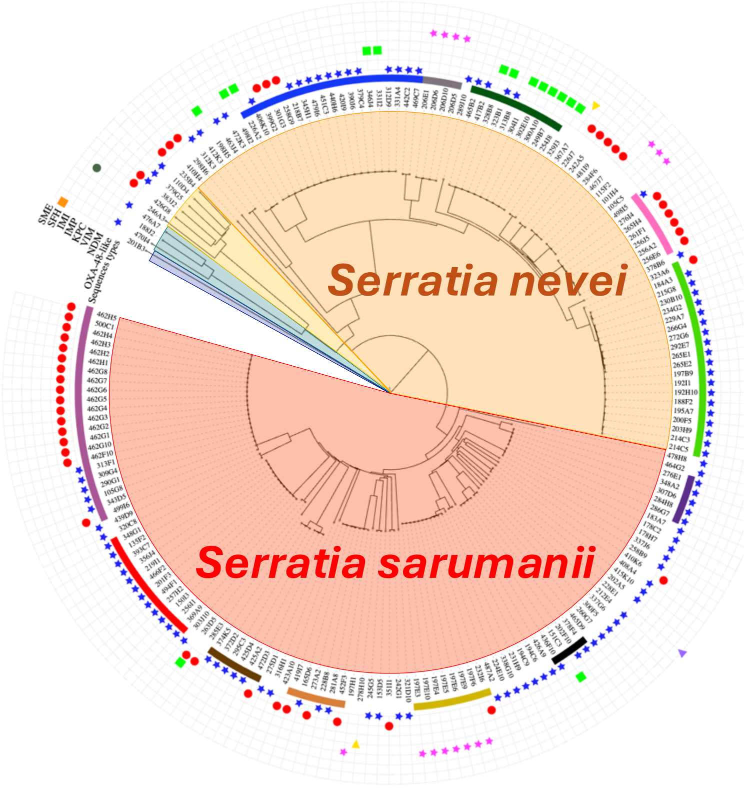

Supplement: Graphical abstract.pdf [file TEMI_A_2671515_SM3329.pdf]
